# Single-incision technique for the internal fixation of distal fractures of the tibia and fibula: a combined anatomic and clinical study

**DOI:** 10.1007/s00402-013-1856-9

**Published:** 2013-10-13

**Authors:** Baoqing Yu, Gan Huang, Josiah T. George, Wenrui Li, Sihua Pan, Haiyan Zhou

**Affiliations:** 1 Department of Orthopaedics, Shanghai Pudong Hospital, Fudan University Pudong Medical Center, Shanghai, 201399 China; 2 Department of Orthopaedics, Changhai Hospital, The Second Military University, Shanghai, 200433 China; 3 Department of Orthopaedics, JFK Medical Hospital, Monrovia, Liberia; 4 Department of Orthopaedics, Shaoguan Hospital, Shaoguan, Guangdong China

**Keywords:** Fracture, Distal tibia, Distal fibula, Anatomy, Incision

## Abstract

**Objectives:**

To present a novel single anterior-lateral approach for the treatment of distal tibia and fibula fracture via anatomical study and primary clinical application in order to minimize soft tissue complications.

**Design:**

Both a gross anatomic cadaver and retrospective studies of the single-incision technique in patients recruited between June 2004 and January 2010.

**Setting:**

Level I trauma center.

**Patients/participants:**

Twenty-six legs of 14 adult human cadavers and clinical recruitment of 49 patients (29 males, 20 females) with a mean age of 37.6 years (range 11–68) with fracture of distal 1/3 tibia and fibula.

**Intervention:**

A single anterior-lateral incision technique for open reduction and internal fixations of distal tibia and fibula fractures.

**Main outcome measures:**

To identify the anatomic structures at risk in the anterolateral aspect of the lower leg and explicit the safe dissection distance from the extensor digitorum longus (EDL) to tibia and fibula, 26 legs of 14 adult human embalmed specimens were recruited in the anatomical study with the distance between the EDL and the anterior edge of the distal thirds of the tibia, as well as the distance between the EDL and the anterior edge of the distal thirds of the fibula were measured, and their mutual relationships to the surrounding anatomical structures described. Mean average standard deviations were also calculated. As for the clinical study, the quality of bone union and soft tissue healing were noted.

**Results:**

The mean distances between the distal tibia and the EDL were measured to be 2.96 ± 0.46 cm (proximal), 1.85 ± 0.25 cm (middle), and 2.15 ± 0.30 cm (distal), and that between the fibula and the EDL were 1.82 ± 0.28 cm (proximal), 2.09 ± 0.31 cm (middle), and 2.30 ± 0.27 cm (distal), which means the safe gap from the distal tibia to EDL was1.6–3.4 cm and from the EDL to fibula was 1.5–2.6 cm. The anterior tibial vein and artery and the deep fibular nerve lie on the anterior interosseous membrane over the lateral surface of the distal tibia were excellently visualized. Review of clinical outcomes in 49 patients with combined distal tibial and fibular fractures who underwent reduction and fixation with the single-incision technique, revealed uneventful fracture healings in 47 patients; and two cases of superficial wound necrosis which were treated and healed in 4 months. There was no case of delayed union or non-union.

**Conclusion:**

Distal fibula fracture occurring with distal tibia fracture poses a challenge for stable fixation. This has necessitated the need for dual incisions on the distal leg to approach each fracture for reduction and fixation. However, a single anterolateral incision enables the safe approach to the lateral aspects of the distal tibia and fibula thus eliminating the need for two separate incisions and minimizing the soft tissue complication to some extent. Meanwhile, the neurovascular bundle at risk during operation, distal tibia and fibula is clearly exposed in the single anterior-lateral incision.

## Introduction

Distal tibia extra-articular fractures sustained from high-energy trauma commonly involve fibular fractures and soft tissue injury. High-energy axial compressive force, direct bending or low-energy rotational forces which result from motor vehicle accident, fall from height, or twisting injuries, account for the common causes of distal tibial and fibular fractures. Due to the type, etiology, limited soft tissue covering and blood supply, these fractures often result to non-union and soft tissue complications [1–4].

The treatment goal of tibial fixation is to maximize fracture stability without increasing soft tissue morbidity from surgical intervention. This poses a challenge for most distal tibial fractures [5]. Recommendation for surgical treatment involves open reduction and internal fixation [6, 7], external fixation [8] and intramedullary nailing [5, 9, 10]. When rigid fibular fixation is required, open reduction and internal fixation generally involve two separate incisions: a lateral incision to approach the fibula and an anterolateral incision to approach the tibia [11]. These combined incisions can cause extensive soft tissue dissection and periosteal injury, with a subsequently high risk of deep infection and skin necrosis [12–14].This is partially true if the initial injury involved more extensive soft tissue damage [15] and if the two incisions during surgery are not separated by a distance of 7 cm or more [16].

Recently, minimally invasive plate osteosynthesis (MIPO) has been proposed as an alternative technique for the preservation of soft tissue at the fracture site. However, if distal tibial fracture cannot reduce prior to surgery by manual traction or with the help of a distracter, MIPO technique should not be considered [17, 18].

This combined efforts of cadaver anatomic and clinical studies seek to first describe the relationship between the distal tibia and fibula and their regional muscular, and neurovascular structures, and secondly prove the high safety and benefits with the use of a single anterolateral incision on the distal leg for the fixation of both the fibula and tibia to avoid severe soft tissue damage and related complications.

## Materials, patients and methods

### Anatomical study

The cadaver anatomical study was performed on 26 legs of 14 adult human embalmed specimens; all legs had normal external appearances and no macroscopic evidences of previous trauma or degenerative changes. Investigations were conducted on both legs in 12 cadavers; and on a single leg in two cadavers (1 right, 1 left) because the contralateral legs were not suitable for study. There were nine male and five female cadavers involved; with an average age of 53 years (range 42–71 years) at the time of death. All measurements were made with the leg in the spontaneous extended position, and an average plantar flexion of 30°.

**Fig. 1:**
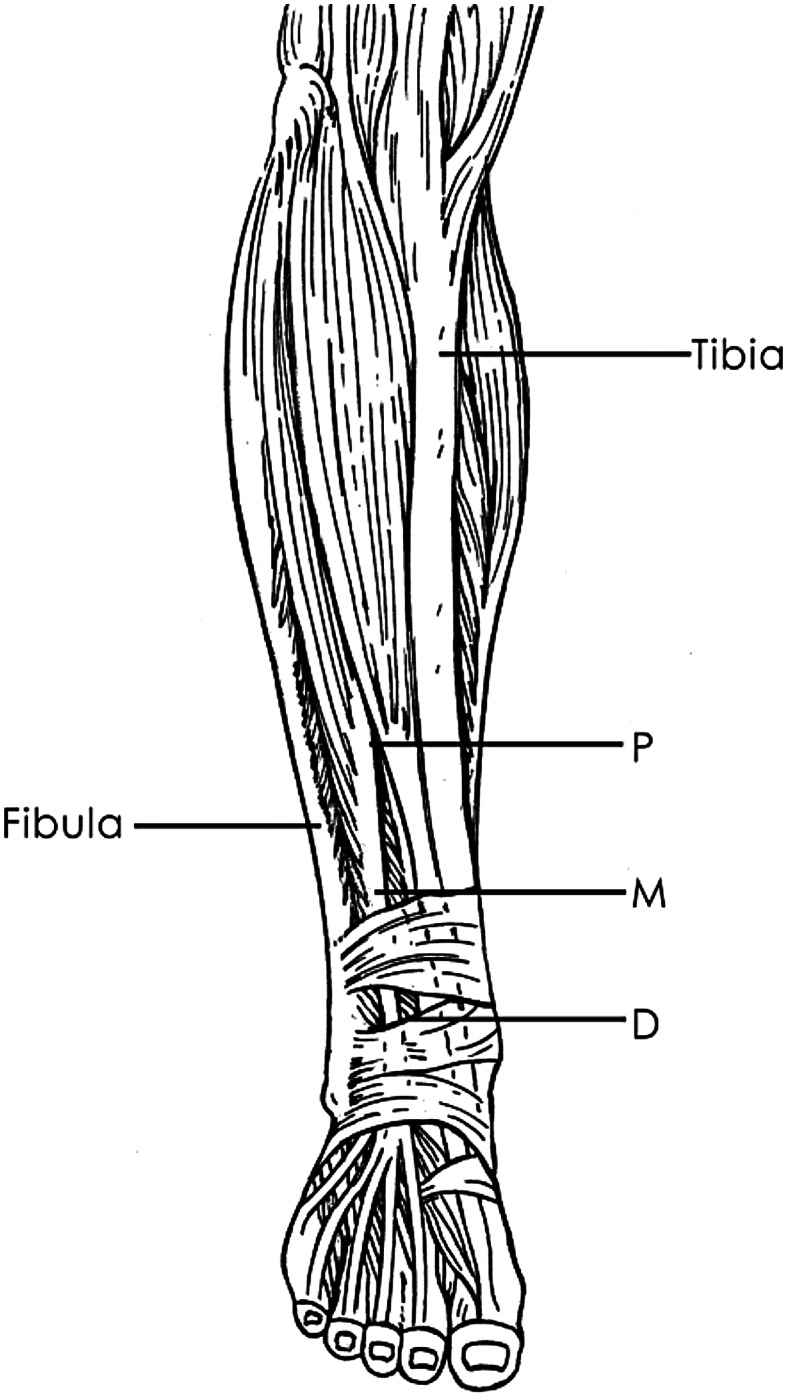
Measurement diagram of the distance between the extensor digitorum longus (EDL) and the tibia and fibula. *P* proximal measurement point of the distal third of the lower leg, *M* the middle measurement point, *D* the distal measurement point

Cadaver dissections were carried out using the technique as follows: Through an anterolateral incision made on the distal leg, the superficial and deep fascial layers were exposed. The anterior crural compartment was opened in the midline. Each of the muscles of the anterior compartment was dissected including tibialis anterior (TA), the extensor hallucis longus (EHL), the extensor digitorum longus (EDL), and the peroneus longus and peroneus brevis. The superficial peroneal nerve was observed as it coursed over the junction of the lower third of the lower leg through the deep fascia and into the subcutaneous tissue between peroneus longus and peroneus brevis muscles. Anterior to the interosseous membrane, the anterior tibial nerve and artery and the deep fibular nerve rested upon the lateral surface of the distal tibia. Following these anatomic identifications, the lower leg lengths were measured from the tip of the fibula head to the tip of the lateral malleolus. Measurements were taken by the distance from the proximal, middle and distal thirds, of the tibia and fibula to the EDL (Fig. 1). The distance between the EDL and the anterior edge of the fibula was measured (accuracy value, 0.01 mm).

**Fig. 2:**
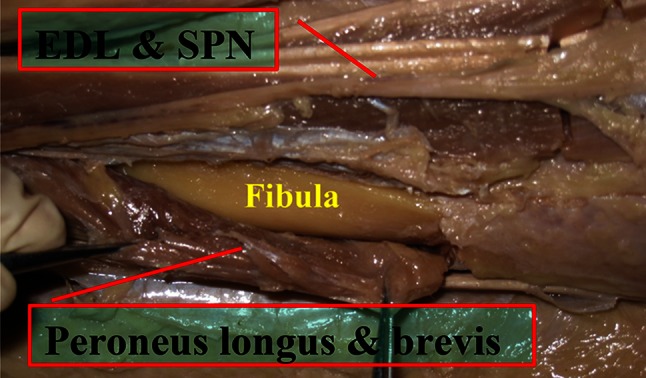
The extensor digitorum longus (EDL) and superficial peroneal nerve (SPN) retracted medially, and peroneus longus and peroneus brevis retracted laterally to expose the fibula

**Fig. 3:**
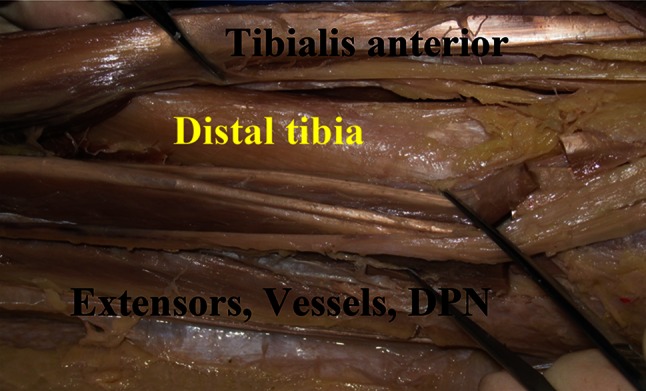
The tibialis anterior retracted medially and the extensor hallucis longus (EHL), extensor digitorum longus (EDL), anterior tibial artery and vein, and deep peroneal nerve (DPN) retracted laterally to expose the tibia

Finally, exposure of the distal third of the fibula was accomplished by retracting the EDL muscle medially and retracting the peroneus longus and peroneus brevis muscles laterally (Fig. 2). The distal third of the tibia was exposed by retracting the TA muscle medially while the EHL, EDL, the anterior tibial vein and artery, and the deep fibular nerves were being retracted laterally through the same anterolateral incision (Fig. 3). Subsequently, the average values from measurements in the legs were calculated. Statistical analysis was carried out with the use of SPSS for windows 11.0 version.

### Clinical implementation study

Between June 2004 and January 2010, there were 49 patients (29 males, 20 females) with a mean age of 37.6 ± 11.2 years (range 18–68 years), who presented with fracture of the distal third of the tibia and fibula, in which the fibula required fixation. Inclusion criteria were the metaphyseal fracture location with or without intra-articular extension into the ankle joint. Exclusion criteria were diaphyseal fractures, nonreconstructable pilon fractures requiring fine-wire external fixation. All the patients were treated with the single anterolateral incision as described above. All fractures were suitable for open reduction and internal fixation. The etiologies of the fractures were as follows: direct forces on 37 cases, indirect forces on 12 cases. 25 cases, motor vehicle accident; 16 cases, fall from heights; and 8 cases, sports injury. There were 11 cases of opened tibia fracture, while there were 38 cases of closed fracture. Based on the AO classification system, modified by Muller, the cases are categorized as follows: 42-A1, 12 cases; 42-A2, 8 cases; 42-B1, 18 cases; and 42-C1, 11 cases.

## Operative technique

After induction of anesthesia, the patient is placed in the supine position with a bump placed underneath the ipsilateral hip to prevent the usual external rotation of the limb. The operative limb extended at the knee joint, prep and draped in the appropriate fashion. The limb is later exsanguinated and tourniquet inflated to reduce blood flow to the surgical site.

**Fig. 4:**
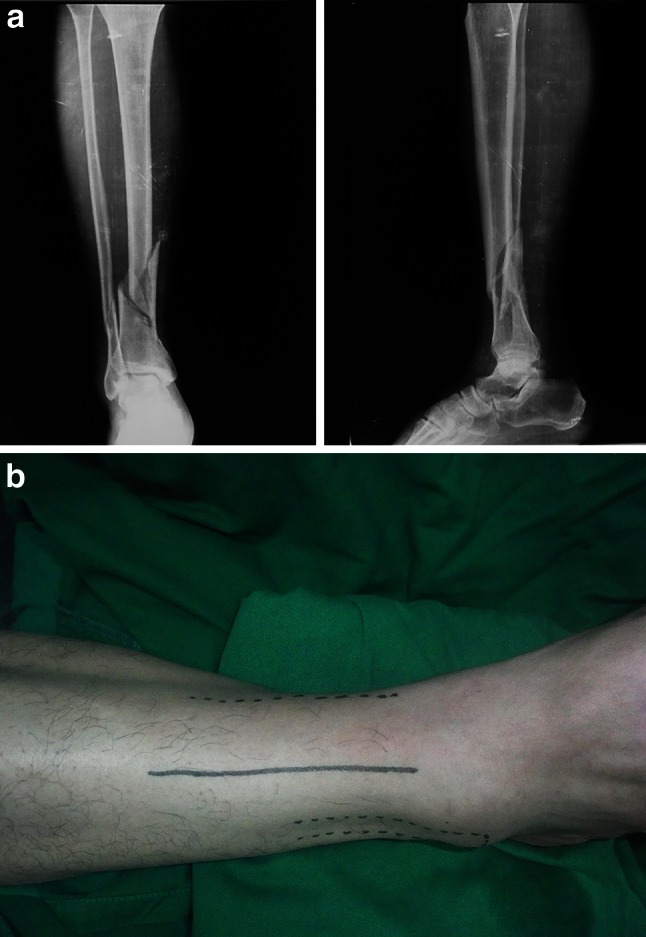
**a** X-ray of a 42-C1 fracture of the right leg. **b** Superficial markings of prominent anatomic features for the longitudinal incision made along the line of projection of the EDL muscle

**Fig. 5:**
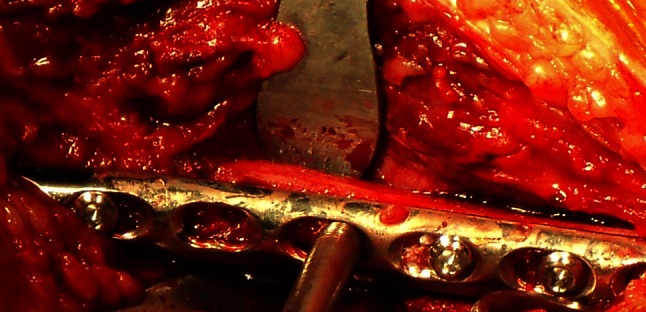
Fixation of the fibular fracture following retraction of the extensor digitorum longus muscle medially and retraction of the peroneus longus and peroneus brevis muscles laterally

A longitudinal anterolateral incision is made on the skin and subcutaneous tissues on the distal leg along the projection line of the extensor digitorum longus muscle (Fig. 4a, b) between the tibia and fibula; the incision was centered on the fracture. The distal end of the incision can generally be extended to about 2 cm above the tibial articular surface without exposure of the subtalar joint. The proximal end of the incision can also be further extended depending on the nature of the fibular fracture. Following identification of the anatomic structures and blunt dissection, the fibula fracture is first to be exposed and fixed so as to get the length reference of the tibia [19] (Fig. 5).

**Fig. 6:**
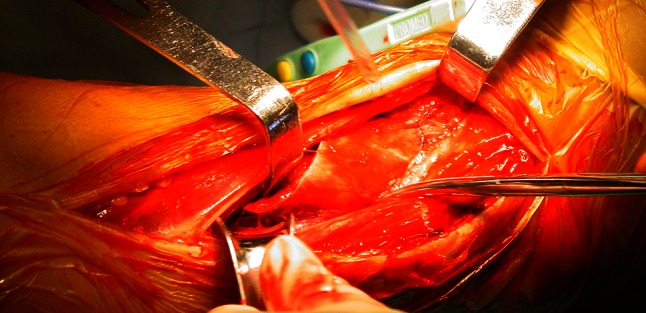
Tibialis anterior muscle retracted medially and EHL, EDL, anterior tibial vein and artery and the deep fibular nerve retracted laterally for exposure of tibia

**Fig. 7:**
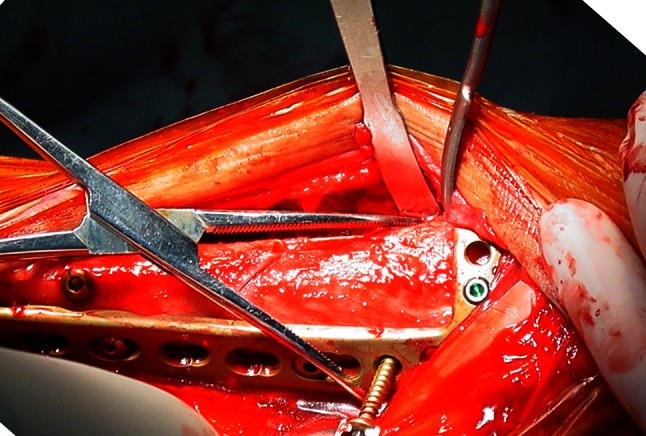
The fixation of the distal tibia fractures. The anatomic structure, distal tibia and fibula are clearly exposed in the incision

And then the extensor hallucis longus (EHL) muscle, EDL, the anterior tibial vein and artery, and the deep fibular nerve together being retracted laterally, the tibialis anterior muscle retracted medially to expose the distal tibia (Fig. 6). When reduction of the tibial fracture is achieved, a plate is applied on the lateral aspect of the tibia for fixation (Fig. 7). In a few cases, depending on the fracture type, a buttress plate can be inserted percutaneously on the medial aspect of the leg following reduction. In situations of defect created by the reduction of the fracture fragments in comminuted distal tibial fractures, bone grafts or bone substitute is inserted to fill the void before plate application and fixation.

Post-operatively, the fractured leg is protected by a below knee cast for 3–6 weeks depending on the bone quality and the type of fixator used. Mobilization of the ankle and permission to weight-bear is determined in individual case based on an assessment of pain levels, local edema, radiography, and the degree of metaphyseal–diaphyseal stability obtained at the time of fixation. All patients are followed up at least until full weight-bearing and healing of the soft tissue (median, 10 months; range 6–12 months). During follow-up, the patients are asked to visit the outpatient clinic at 4 weeks, 3 months, 6 months, and 1 year intervals after surgery. All patients are reviewed with post-op X-rays by an orthopedic surgeon on each clinic visit.

## Results

During cadaver anatomic study, the superficial peroneal nerve and deeper neurovascular structures at risk during exposure were clearly visualized in all specimens, every structure from superficial to deep layers was usually undamaged during dissection. Care was also taken while accessing the distal fracture sites to leave them undamaged. The lower leg length of each subject and the distance between the EDL muscle and the tibia and fibula of each specimen are shown in Table 1.

**Table 1:** Distances between the proximal, middle, and distal parts of the distal third of leg (mm)

	Proximal distance ($\bar{X}$ ± S)	Middle distance ($\bar{X}$ ± S)	Distal distance ($\bar{X}$ ± S)
Distance between EDL and tibia (cm)	2.96 ± 0.46	1.85 ± 0.25	2.15 ± 0.30
Distance between EDL and fibula (cm)	1.82 ± 0.28	2.09 ± 0.31	2.30 ± 0.27

*EDL* extensor digitorum longus muscle

**Fig. 8:**
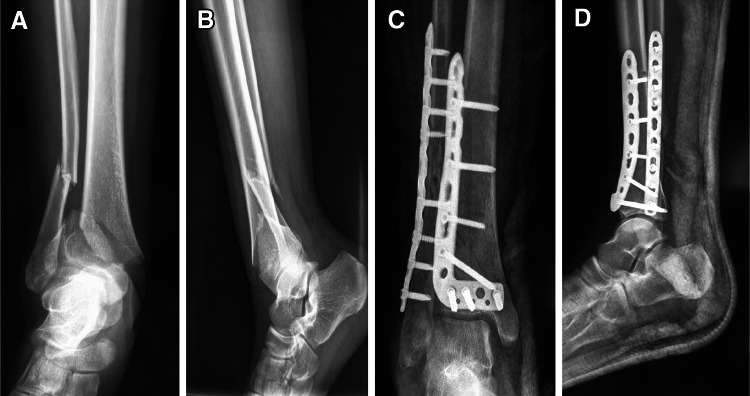
A 45-year-old man was involved in a car crash sustained a closed distal tibia-fibula fracture, the surgery was performed 10 days post-injury with the single anterior-lateral approach. **a** AP radiograph, **b** lateral radiograph, **c** after the fixation of plates via the single anterior-lateral incision, AP radiograph and **d** lateral radiograph

During the study period, of the 49 patients who presented with combined distal tibial and fibular fractures, with the mean arrival time of 6.3 ± 3.7 h (range 1–12 h); surgery was performed within 24 h after the trauma in 20 patients; within 3 days in 7 patients; and within 4–7 days in 19 patients. The mean duration of surgery was 81 min (range 55–110 min). The achieved fracture reductions were anatomical or near anatomical without angular displacement in all cases. Of these cases, fibular fixation was conducted first in all patients, which was followed by tibial reduction under direct vision using the same incision, with the plate applied laterally on the tibia and fibula (Fig. 7). Recovery in 47 out of 49 patients was uneventful while the two cases of superficial necrotic wounds which were treated and healed in 4 weeks. Four cases later reported peripheral neuropathy in the dorsum of the foot but had no influence on the function of affected limbs. There was no non-union or delayed union in any case. The mean bone healing time was 10.6 weeks (10–12 weeks) (Fig. 8). At the last follow-up visits the fractures were in excellent positions and healed radiographically, and the patients walked unassisted and without pain. These fractures were therefore considered healed.

## Discussion

Treatment of fracture of the distal third of the tibia and fibula can be challenging. A wide range of modalities exist from closed reduction to the use of the external fixator. The major objectives in the treatment of these fractures are rapid and optimal healing, minimization of any soft tissue complication and loss of function, and the prevention of non-union or malunion [3]. The most significant cause of delayed union or non-union in cases that undergo plate fixation is the compromising of the circulation to the distal tibia, which is already damaged after the fracture, even with the use of bone graft [10, 20]. The two cases of superficial wound necrosis reported were observed to be the result of early and aggressive operative procedures as the skin covering did not fully recover before intervention. The appropriate mechanisms were employed to ensure full tissue recovery. The peripheral neuropathy also reported for four patients was due to excessive and prolonged traction of soft tissues during fracture reduction and plate fixation. These symptoms resolved with time.

Open reduction and internal fixation, in addition to MIPO, are two alternative techniques that have been used to preserve the surrounding soft tissue and prevent delayed union and non-union. When the fixation of both tibia and fibula is required, open reduction and internal fixation generally involves two separate incisions, which can lead to extensive soft tissue dissection and periosteal injury. This technique risks result to delayed union or non-union of distal tibial fractures, even in the most expert hands. MIPO technique should not be considered if distal tibial fracture cannot reduce while closed.

In 2000, Sanagaram et al. [5] proposed the single-incision treatment for the management of these fractures, and reported a good clinical efficacy. However, the anatomical basis of an anterolateral approach to the distal tibia and fibula has not been discussed. Our large study series of cadaver lower limbs, in order to define the anatomy of distal tibia and fibula has optimized our improved technique and outcomes. The dissections demonstrated an obvious muscle gap and nerve interface in the anterolateral lower leg; an incision made in this region could avoid the major blood vessels and nerves, including the superficial peroneal nerve between the peroneus longus and peroneus brevis in the superficial layers injured. In the deep structures, the course of the anterior tibial artery and nerve and the deep peroneal nerve run along the outer surface of the distal tibia; they are generally not easy to be damaged by retracting laterally for protection under direct vision. In addition, the dissection demonstrated that during the exposure of the tibia, the TA, EDL, and EHL muscles needed to be retracted medially and laterally to create a gap between 1.6 and 3.4 cm. While exposing the fibula, the gap between the EDL and the peroneus longus and peroneus brevis only needed to be retracted by between 1.5 and 2.6 cm.

It is known that the distal tibia has less vascular and soft tissue support than any other part of the tibia. Infections are therefore a relatively common complication [21, 22]. It is also known that open reduction and plate fixation of a traumatized extremity increases soft tissue damage and the risk of compartment syndrome [3]. The use of a single incision is minimally invasive to the soft tissue and opens the anterior compartment of the leg, which has the combined benefit of eliminating the risk of compartment syndrome.

Clinically, 24 fractures treated with the single anterolateral incision technique for distal tibial and fibular fractures, healed with the normal time period and there was one delayed union (4 %). There were no cases of malunion or non-union. Though two cases had suffered from soft tissue complication, whom were healed in 4 months after the initial surgery. These results were consistent with those of preliminary study conducted by Shantharam et al. [5]. The minimal effect on the periosteum, vascular supply to the bone, complete stabilization of the distal bony fragment and accurate reduction of fractures are the major advantages of the single-incision technique, which minimizes complications of delayed union, malunion and non-union.

## Conclusion

Antecedent by cadaver study, the single-incision technique is a safe and useful choice for the treatment of distal tibial and fibular fractures. This technique aims to reduce tissue trauma, preserve the periosteal vascular integrity, and aid fracture reduction with the use of the MIPO technique. In light of the results of this study, we believe that the single anterolateral incision technique may be used as an alternative method in the treatment of distal fractures of the tibia and fibula. However, due to the small number of patients involved in our study, further research is needed to establish the safety and efficacy of this single-incision technique in the long term.

## References

[1] Taylor JC, Crenshaw AH (1992). Fractures of the lower extremity. Campbell’s Operative Orthopaedics.

[2] Trafton PG, Bray TJ, Simpson LA, Browner BD, Jupiter JB, Levine AM, Trafton PG (1992). Fractures of soft tissue injuries of the ankle. Skeletal Trauma.

[3] Oh C-W, Kyung H-S, Park I-H (2003). Distal tibia metaphyseal fractures treated by percutaneous plate osteosynthesis. Clin Orthop.

[4] Helfet DL, Suk M (2004). Minimally invasive percutaneous plate osteosynthesis of fractures of the distal tibia. Instr Course Lect.

[5] Shantharam SS, Naeni F, Wilson EP (2000). Singleincision technique for internal fixation of distal tibia and fibula fractures. Orthopedics.

[6] Mosheiff R, Safran O, Segal D (1999). The unreamed tibial nail in the treatment of distal metaphyseal fractures. Injury.

[7] Gorczyca JT, McKale J, Pugh K (2002). Modifiedtibial nails for treating distal tibia fractures. J Orthop Trauma.

[8] Megas P, Zouboulis P, Papadopoulas AX (2003). Distal tibial fractures and non-unions treated with shortened intramedullary nail. Int Orthop.

[9] Ozsoy MH, Tuccar E, Demiryurek D (2009). Minimally invasive plating of the distal tibia: do we really sacrifice saphenous vein and nerve? A cadaver study. J Orthop Trauma.

[10] Mirza A, Moriarty AM, Probe RA (2010). Percutaneous plating of the distal tibia and fibula: risk of injury to the saphenous and superficial peroneal nerves. J Orthop Trauma.

[11] Puno RM, Teynor JT, Nagano J (1986). Critical analysis of results of treatment of 201 tibial shaft fractures. Clin Orthop Rel Res.

[12] Trafton PG (1988). Closed unstable fractures of the tibia. Clin Orthop Rel Res.

[13] Oh CW, Kyung HS, Park IH (2003). Distal tibia metaphyseal fractures treated by percutaneous plate osteosynthesis. Clin Orthop Rel Res.

[14] Zelle BA, Bhandari M, Espiritu M (2006). Treatment of distal tibia fractures without articular involvement: a systematic review of 1125 fractures. J Orthop Trauma.

[15] Kellam JF, Waddell JP (1979). Fractures of the distal tibial metaphysis with intraarticular extension: the distal tibial explosion fracture. J Trauma.

[16] Pugh KJ, Wolinsky PR, McAndrew MP (1999). Tibial pilon fractures: a comparison of treatment methods. J Trauma.

[17] Anglen JO (1999). Early outcome of hybrid external fixation for fracture of the distal tibia. J Orthop Trauma.

[18] Gl Im, Tae SK (2005). Distal metaphyseal fractures of tibia: a prospective randomized trial of closed reduction and intramedullary nail versus open reduction and plate and screws fixation. J Trauma.

[19] Brumback RJ, Mcgarvey WC (1995). Fractures of the tibial plafond. Orthop Clin N Am.

[20] Manninen MJ, Lindahl J, Kankare J (2007). Lateral approach for fixation of the fractures of the distal tibia. Outcome of 20 patients. Technical note. Arch Orthop Trauma Surg.

[21] Brumback RJ, Levine AM (1996). Fractures of the tibial plafond. Ortlaopaedic Knowledge Update: Trauma.

[22] Miiller ME, Allgower M, Schneider R (1991). Manual of Internal Fixation.

